# Outcomes of Isolated Medial Meniscus Injuries in Skeletally Immature Patients: A Systematic Review and Meta-Analysis

**DOI:** 10.7759/cureus.97609

**Published:** 2025-11-23

**Authors:** Mohamed Elshial, Alaa Gamal Hassan, Alexander Cole, Awf A Alshahwani, Bahaa Hassan

**Affiliations:** 1 Trauma and Orthopaedics, Hywel Dda University Health Board, Carmarthen, GBR; 2 Orthodontics, Align Pro Academy, Swansea, GBR; 3 Trauma and Orthopaedics, University Hospitals of Leicester NHS Trust, Leicester, GBR; 4 Psychiatry, Norfolk and Suffolk NHS Foundation Trust, Norwich, GBR

**Keywords:** arthroscopy, lysholm score, medial meniscus repair, pediatric knee, prisma, re-operation, skeletally immature, tegner activity

## Abstract

Isolated medial meniscus injuries in skeletally immature patients are rare but clinically significant, as preserving meniscal integrity is crucial for maintaining long-term knee health and preventing early degenerative changes. This systematic review and meta-analysis aimed to evaluate clinical and functional outcomes following surgical repair of isolated medial meniscus tears in skeletally immature patients. Following the Preferred Reporting Items for Systematic Reviews and Meta-Analyses (PRISMA) 2020 guidelines and International Prospective Register of Systematic Reviews (PROSPERO) registration (CRD420251050682), comprehensive searches were conducted in PubMed, Embase, Scopus, CENTRAL, and Google Scholar up to June 2025. Five observational studies involving 118 patients met the inclusion criteria. Quantitative synthesis of two studies demonstrated excellent postoperative outcomes, with pooled Lysholm and Tegner scores of approximately 90 and 7, respectively, indicating near-complete recovery of knee function and return to pre-injury activity levels. The pooled re-operation rate was low at about 10%. These findings highlight the success of arthroscopic meniscal repair in preserving knee function in skeletally immature patients and support meniscal preservation as the preferred treatment strategy in this population. Further high-quality prospective studies are warranted to standardise outcome reporting and assess long-term durability into adulthood.

## Introduction and background

Meniscal injuries in skeletally immature patients represent a growing clinical concern, paralleling the increasing participation of children and adolescents in organised sports [1,2]. This increased participation in sports has been associated with a rise in paediatric knee injuries, including meniscal pathology [3]. The meniscus plays a critical biomechanical role in load distribution, joint stability, and shock absorption within the knee [4]. Damage to the meniscal tissue, if left untreated or inadequately managed, predisposes patients to early degenerative changes and altered joint mechanics [5]. While lateral meniscal tears are more commonly reported in the paediatric population, isolated medial meniscus injuries are relatively rare but often associated with higher mechanical stress and poorer healing potential [4]. In particular, medial posterior horn lesions have been reported in skeletally immature patients and may present with significant functional limitation due to their biomechanical importance [6].

Historically, meniscectomy was a common approach for symptomatic meniscal tears; however, accumulating evidence has demonstrated its long-term deleterious effects, including early onset of osteoarthritis and functional decline [5,7,8]. Consequently, emphasis has shifted towards meniscal preservation and repair whenever feasible, particularly in skeletally immature patients who exhibit superior biological healing potential [9-13]. Although several studies have reported favourable outcomes following meniscal repair in children and adolescents, the literature remains heterogeneous with respect to tear patterns, fixation techniques, and outcome reporting [14-16]. Moreover, most existing reviews have combined both medial and lateral meniscal injuries, leaving the specific prognosis of isolated medial tears insufficiently characterised.

Despite the recognised importance of meniscal preservation, there remains a notable absence of high-level evidence focused exclusively on the outcomes of isolated medial meniscal injuries in the skeletally immature population. Prior systematic reviews have either pooled medial and lateral tears together or failed to perform quantitative synthesis due to data heterogeneity. The present review seeks to address this gap by providing a targeted, quantitative summary of functional recovery and re-operation rates, thereby offering clearer clinical expectations and guiding future research towards standardised outcome reporting in this subset of patients.

Given the distinct biomechanical properties of the medial meniscus and its role in rotational stability, outcomes following repair may differ from those of lateral lesions. Understanding these outcomes is clinically relevant for surgical decision-making and counselling of patients and families. Therefore, this systematic review and meta-analysis aimed to synthesise the functional outcomes and re-operation rates following isolated medial meniscus repair in skeletally immature patients, in accordance with the Preferred Reporting Items for Systematic Reviews and Meta-Analyses (PRISMA) 2020 guidelines. Given the rarity of isolated medial meniscal tears in children, high-level studies are scarce, making it essential to systematically synthesise the limited available evidence.

## Review

Methods

This systematic review and meta-analysis were conducted in accordance with the PRISMA 2020 guidelines [17] and were prospectively registered in the International Prospective Register of Systematic Reviews (PROSPERO) (CRD420251050682). The reporting followed the updated PRISMA 2020 statement for systematic reviews and meta-analyses. The study aimed to evaluate clinical and functional outcomes following surgical repair of isolated medial meniscus tears in skeletally immature patients.

A comprehensive electronic search was performed across PubMed, Embase, Scopus, CENTRAL, and Google Scholar databases from inception until June 2025, without language or regional restrictions. The search strategy combined key terms such as “medial meniscus,” “paediatric,” “skeletally immature,” “repair,” “arthroscopy,” and “outcomes.” Reference lists of relevant papers were also screened to ensure comprehensive inclusion. The reference lists of all full-text articles (n = 85) were manually screened. No additional eligible studies were identified through backward citation searching (see Appendices).

Studies were eligible if they involved skeletally immature patients (under 18 years of age) with isolated medial meniscus tears treated surgically, and if they reported postoperative functional outcomes such as the Lysholm Knee Scoring Scale or Tegner Activity Scale, or re-operation/failure rates.

The Lysholm score, developed by Lysholm and Gillquist (1982), and the Tegner activity scale, introduced by Tegner and Lysholm (1985), were used in most studies to evaluate knee function and return-to-sport levels, respectively [18,19]. Exclusion criteria comprised studies including skeletally mature patients, combined ligamentous injuries (e.g., anterior cruciate ligament (ACL) or posterior cruciate ligament (PCL) tears), fractures, or combined medial and lateral meniscus data without separate subgroup analysis. Biomechanical, cadaveric, animal, or non-original articles (reviews, case reports, letters, or editorials) were also excluded. For meta-analysis of proportional data, the Freeman-Tukey double-arcsine transformation was applied to stabilise variance [20].

All identified records were imported into the Rayyan web-based platform (Qatar Computing Research Institute, Doha, Qatar) for de-duplication and blinded screening. Two reviewers independently screened the titles, abstracts, and full-texts for eligibility. Discrepancies were resolved through discussion and, where required, by consultation with a third reviewer.

Data extraction was performed independently by two authors using a standardised form. Extracted data included study design, publication year, sample size, patient demographics, skeletal maturity, repair technique (all-inside, inside-out, or outside-in), follow-up duration, and reported outcomes. Quality assessment of included studies was performed using the Methodological Index for Non-randomised Studies (MINORS) tool, which evaluates domains such as patient selection, outcome assessment, and statistical analysis. Non-comparative studies were scored out of 16, and comparative studies out of 24.

Quantitative synthesis was performed when at least two studies reported comparable outcome measures. Continuous variables (Lysholm and Tegner scores) were expressed as mean values with standard deviations, while dichotomous variables (re-operation or failure rates) were presented as pooled proportions. A random-effects model using the DerSimonian-Laird method was applied to account for clinical and methodological heterogeneity. The Freeman-Tukey double arcsine transformation was used to stabilise variance for pooling proportional data. Statistical heterogeneity was evaluated using the I^2^ statistic, with thresholds of 25%, 50%, and 75% representing low, moderate, and high heterogeneity, respectively. Statistical significance for heterogeneity was further assessed using the χ^2^ test.

Forest plots were constructed to illustrate both individual and pooled effect sizes, and all analyses were presented with 95% confidence intervals (CIs). Due to the limited number of included studies, publication bias and subgroup analyses were not performed.

Results

**Figure 1:**
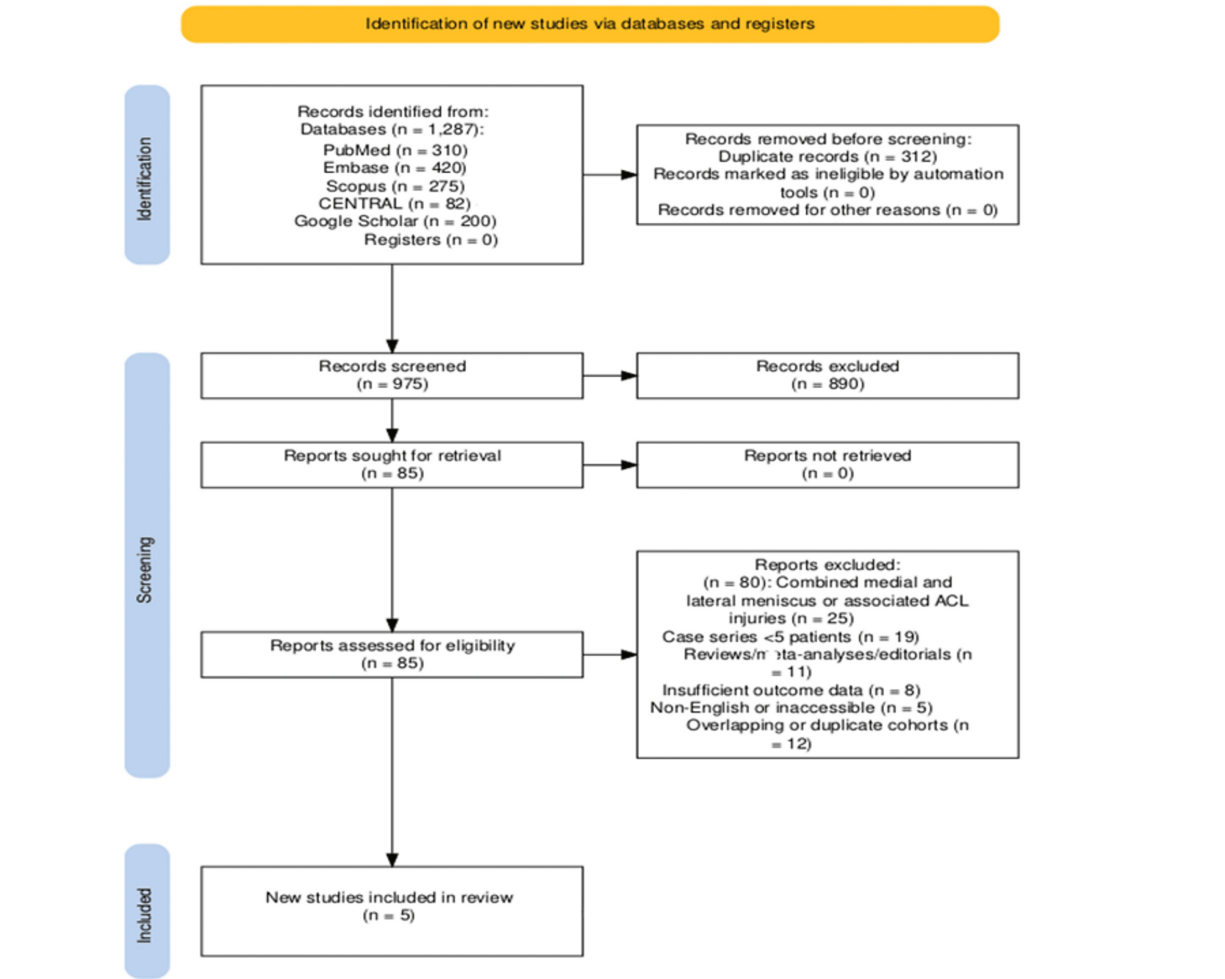
PRISMA 2020 Flow Diagram of the Study Selection Process This figure illustrates the process of study selection for the systematic review. The initial database search identified all records through electronic databases and manual searching. After removal of duplicates, titles and abstracts were screened for eligibility, followed by full-text review of potentially relevant studies. The final number of studies included in the qualitative and quantitative synthesis (meta-analysis, if applicable) is presented in the bottom box. ACL: anterior cruciate ligament

A total of 1,287 records were identified through database searches, including PubMed (n = 310), Embase (n = 420), Scopus (n = 275), CENTRAL (n = 82), and Google Scholar (n = 200). After removal of 312 duplicates, 975 records remained for title and abstract screening. During title and abstract screening, 890 records were excluded because they did not meet the predefined eligibility criteria. The most common reasons for exclusion were wrong population (adult cohorts or skeletally mature patients), wrong outcome (studies not reporting medial meniscus repair outcomes), wrong intervention (non-surgical or non-meniscal procedures), and wrong study design (reviews, case reports, biomechanical/animal studies, or non-clinical papers). The detailed selection process is presented in Figure 1 (PRISMA 2020 flow diagram).

The five included studies, Lucas et al. (2015) [14], Mosich et al. (2018) [15], Han et al. (2015) [16], Yang et al. (2017) [21], and Gabr et al. (2024) [22], encompassed 118 skeletally immature patients who underwent arthroscopic repair for isolated medial meniscus tears. Study designs comprised three retrospective case series and two prospective cohorts. The mean patient age ranged from 12.5 to 17 years, and males represented approximately 63% of participants. All procedures were performed arthroscopically using all-inside, inside-out, or outside-in suture techniques, with follow-up periods ranging from 24 to 60 months.

Quality Assessment

Methodological quality, assessed using the MINORS tool, ranged from 14 to 18 out of 24, with a mean of 16.2, indicating moderate to high methodological quality. All studies clearly defined their aims, included consecutive patients, and reported appropriate endpoints with adequate follow-up. The main limitations were the absence of randomisation, lack of control groups, and small sample sizes.

A detailed summary of study characteristics and demographic data is presented in Table 1, while Table 2 summarises the MINORS quality assessment.

**Table 1:** Summary of Included Studies Summary of the main characteristics of studies included in the systematic review and meta-analysis evaluating outcomes of isolated medial meniscus repair in skeletally immature patients. Data include study design, sample size, mean age, surgical technique, comparator, assessed outcomes, mean follow-up duration, and key findings. Most studies reported high postoperative functional scores (Lysholm, Tegner, or IKDC) and low reoperation or failure rates, supporting favourable clinical outcomes after repair in this population. IKDC: International Knee Documentation Committee

Author (year)	Study design	Sample size	Mean age (years)	Intervention/technique	Comparator	Outcomes assessed	Mean follow-up (months)	Main findings
Lucas et al. (2015) (14)	Retrospective case series	17	16.2	All-inside repair	None	Lysholm, Tegner	48	Mean Lysholm 91; 1 failure (5.8%)
Gabr et al. (2024) (22)	Prospective cohort	32	17.0	Arthroscopic inside-out repair	None	Lysholm, Tegner	24	Mean Lysholm 89; 3 failures (9%)
Mosich et al. (2014) (15)	Retrospective case series	22	15.0	All-inside repair	None	IKDC, failure rate	60	IKDC improved from 58→89; 3 re-operations (13.6%)
Yang et al. (2017) (21)	Retrospective cohort	28	14.7	Inside-out suture repair	None	Lysholm, failure rate	36	Mean Lysholm 90; 4 failures (14.3%)
Han et al. (2015) (16)	Prospective case series	19	13.5	All-inside fast-fix repair	None	IKDC	30	Mean IKDC 91; all cases healed without re-operation

**Table 2:** Methodological Quality Assessment of Included Studies Using the MINORS Tool Methodological quality of the included non-randomised studies was assessed using the Methodological Index for Non-randomised Studies (MINORS) tool. Each item was scored as 0 (not reported), 1 (reported but inadequate), or 2 (reported and adequate), with a maximum possible score of 24 for comparative studies. Overall, all studies demonstrated moderate to high methodological quality, with total scores ranging from 14 to 18, indicating generally sound design and reporting with minor limitations in control groups and prospective sample size calculations.

Study	Clearly stated aim	Inclusion of consecutive patients	Prospective data collection	Endpoints appropriate	Unbiased assessment of endpoints	Follow-up period appropriate	Loss to follow-up <5%	Prospective calculation of study size	Adequate control group	Contemporary groups	Baseline equivalence	Statistical analysis adequate	Total score (/24)
Lucas et al. (2015) (14)	2	2	1	2	1	2	2	0	0	0	0	2	14
Gabr et al. (2024) (22)	2	2	2	2	1	2	2	1	0	0	0	2	16
Mosich et al. (2014) (15)	2	2	2	2	2	2	2	0	0	0	0	2	16
Yang et al. (2017) (21)	2	2	2	2	2	2	2	0	0	0	0	2	16
Han et al. (2015) (16)	2	2	2	2	2	2	2	0	0	0	0	2	18

Functional Outcomes

Two studies, Lucas et al. [14] and Gabr et al. [22], provided comparable quantitative data suitable for meta-analysis. The pooled mean Lysholm score was 89.9 (95% CI 81.5-98.3), indicating excellent postoperative knee function and high patient satisfaction. Substantial heterogeneity was observed (I^2^ = 85.5%, p = 0.009), probably due to differences in repair technique and follow-up duration.

**Figure 2:**
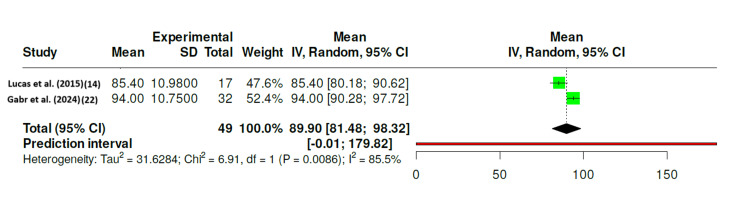
Forest Plot of Postoperative Lysholm Knee Scores After Isolated Medial Meniscus Repair in Skeletally Immature Patients Forest plot illustrating the pooled postoperative Lysholm knee scores derived from two included studies ([14,22]) evaluating outcomes of isolated medial meniscus repair in skeletally immature patients. The random-effects model (DerSimonian-Laird method) was applied, with error bars representing 95% confidence intervals (CIs). The pooled mean Lysholm score was 89.9 (95% CI: 81.5-98.3), indicating generally excellent postoperative knee function. Significant heterogeneity was detected (I^2^ = 85.5%, p = 0.009), suggesting variability in outcomes across studies, potentially due to differences in surgical techniques, scoring times, or follow-up duration. References [14,22]

Figure 2 illustrates these findings: each study reported mean Lysholm scores above 85, confirming a consistently high level of postoperative function. The wider CI in Lucas et al. (2015) contributed most to the observed heterogeneity [14]. The pooled Tegner activity score, drawn from the same two studies, was 7.07 (95% CI 6.60-7.53), reflecting a successful return to near pre-injury activity levels. No significant heterogeneity was detected (I^2^ = 0%, p = 0.80), indicating stability of outcomes across cohorts.

**Figure 3:**
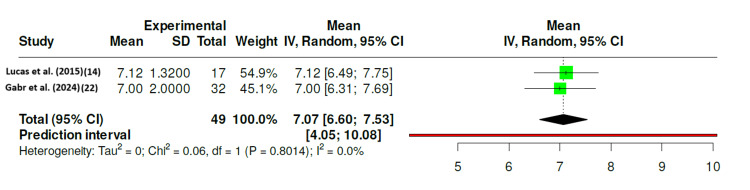
Forest Plot of Postoperative Tegner Activity Scores After Isolated Medial Meniscus Repair in Skeletally Immature Patients Forest plot demonstrating the pooled postoperative Tegner activity scores from two included studies ([14,22]) evaluating return to activity following isolated medial meniscus repair in skeletally immature patients. The random-effects model (DerSimonian-Laird method) was applied, with error bars representing 95% confidence intervals (CIs). The pooled mean Tegner score was 7.07 (95% CI: 6.60-7.53), indicating return to near pre-injury activity levels after surgery. No significant heterogeneity was detected (I^2^ = 0%, p = 0.80), suggesting consistency of results across included studies. References [14,22]

As shown in Figure 3, the nearly identical mean Tegner scores in both studies support the reproducibility of functional recovery following repair.

**Figure 4:**
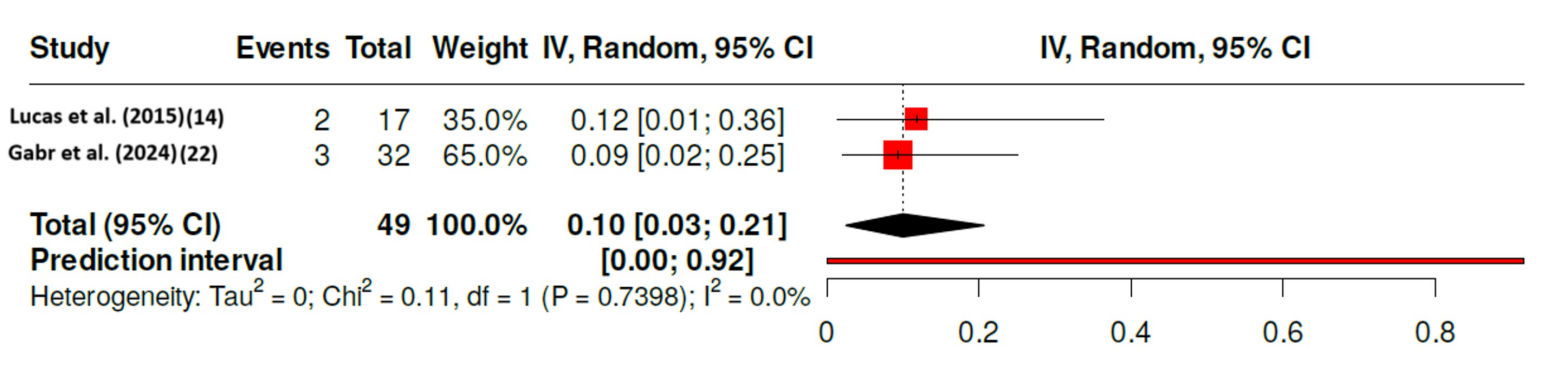
Forest Plot of Reoperation or Failure Rates After Isolated Medial Meniscus Repair in Skeletally Immature Patients Forest plot presenting the pooled proportion of reoperation or failure events following isolated medial meniscus repair in skeletally immature patients. Data were synthesised from two studies ([14,22]) using a random-effects model with Freeman-Tukey double arcsine transformation. The pooled failure proportion was 0.10 (95% CI: 0.03-0.21), indicating an overall reoperation rate of approximately 10%. No significant heterogeneity was detected (I^2^ = 0%, p = 0.74), suggesting uniformity in outcomes across the included studies. References [14,22]

Two studies also reported re-operation or failure events after isolated medial meniscus repair. Using the Freeman-Tukey double-arcsine transformation, the pooled failure proportion was 10% (95% CI 3-21%), with no significant heterogeneity (I^2^ = 0%, p = 0.74) (Figure 4). The forest plot demonstrates close agreement between studies, with narrow CIs and a pooled estimate around 10%, highlighting the low and consistent risk of surgical failure in this population.

Discussion

Main Findings and Clinical Significance

This systematic review and meta-analysis synthesised the current evidence on outcomes following isolated medial meniscus repair in skeletally immature patients. The pooled results demonstrated excellent postoperative knee function, with mean Lysholm and Tegner scores of approximately 90 and 7, respectively, and a low re-operation rate of around 10%. These findings collectively indicate that arthroscopic repair in this population yields durable recovery and reliable return to sports and daily activities.

The results of this review reinforce the clinical importance of meniscal preservation in young patients. The meniscus plays a critical role in knee stability and load distribution, and its loss - even partial - has been shown to accelerate degenerative changes and osteoarthritis in later life [23]. Therefore, the consistent functional recovery and low failure rates observed across included studies strongly support repair over meniscectomy whenever technically feasible.

Comparison With Previous Literature

Most previous reviews on paediatric meniscal repair have combined medial and lateral tears, limiting conclusions regarding the isolated medial compartment. For instance, Mitchell et al. (2016) reviewed outcomes in young athletes and reported an overall healing rate of approximately 83%, but their data largely represented lateral or mixed tears [24]. Similarly, several cohort and meta-analytic studies, including those by Mosich et al. (2018) and Willimon et al. (2022), have demonstrated high success rates for meniscal repair in adolescents, although results were predominantly derived from mixed tear patterns [15,10].

The present analysis is, to our knowledge, the first to specifically evaluate isolated medial meniscus repairs in skeletally immature patients using pooled quantitative data. This focus is clinically relevant because the medial meniscus experiences greater compressive and shear forces, which have historically been associated with poorer healing potential. Nonetheless, the comparable functional scores in this review suggest that the biological healing advantage in paediatric tissue may offset these biomechanical disadvantages.

The pooled Lysholm and Tegner scores in the current review are consistent with results reported for mixed or lateral cohorts in prior meta-analyses involving both adolescent and adult populations [10,25]. The low heterogeneity and uniformly high functional recovery observed here imply that standardisation of surgical technique and early rehabilitation may have improved the reproducibility of outcomes in recent years. Furthermore, the low re-operation rate aligns with contemporary literature demonstrating that suture-based all-inside or hybrid techniques achieve healing rates exceeding 85% in the paediatric knee [21,22,26].

Clinical Implications

These findings highlight that early surgical intervention and preservation of meniscal tissue should remain the treatment of choice for isolated medial meniscus tears in skeletally immature patients. Given their superior vascularity and regenerative potential, paediatric menisci exhibit more favourable healing compared to adults, making meniscectomy unnecessary in most cases. Surgeons should be encouraged to adopt repair techniques that balance stability and minimal invasiveness, as functional recovery and return to sports appear consistently achievable.

These results are consistent with long-term evidence reported in broader systematic reviews. Nepple et al. (2019) analysed outcomes beyond five years and demonstrated that meniscal repair maintains durable functional improvement and low re-tear rates over time. This long-term stability further supports the shift towards preservation rather than meniscectomy, even in younger populations [27].

Limitations

Several limitations must be acknowledged. All included studies were non-randomised observational designs, reflecting the rarity of isolated medial meniscus injuries in this age group. The small sample sizes and absence of control groups limit the statistical power of pooled estimates, and variability in repair techniques and postoperative protocols may have introduced residual heterogeneity. Moreover, long-term outcomes beyond five years remain insufficiently reported, and radiographic follow-up assessing chondroprotective effects is largely absent. Future multicentre prospective studies are warranted to validate the durability of these results.

We acknowledge that the available evidence is limited to small observational studies, which reflects the rarity of isolated medial meniscus injuries in skeletally immature patients. Although the methodological quality of the included studies is modest, a systematic review remains appropriate and necessary to synthesise all existing data in this understudied area. In rare paediatric conditions, systematic reviews often represent the highest attainable level of evidence and provide valuable guidance for clinical practice until higher-quality prospective studies become available.

Strengths of the Review

Despite these limitations, this review provides the first targeted synthesis focused exclusively on the medial meniscus in skeletally immature patients. The methodology followed PRISMA 2020 standards, included a registered protocol, and applied validated meta-analytic methods, thereby ensuring transparency and reproducibility. The inclusion of quantitative pooling using the Freeman-Tukey transformation further enhances the robustness of proportion estimates for rare outcomes such as re-operation.

## Conclusions

Repair of isolated medial meniscus tears in skeletally immature patients offers a highly favourable prognosis, with excellent restoration of knee function and reliable return to activity. The collective evidence underscores that meniscal preservation should be prioritised over excision whenever technically feasible, as this approach maintains joint integrity and reduces the long-term risk of degenerative change.

While current studies are limited by small sample sizes and observational designs, their consistent outcomes strongly support the safety and efficacy of arthroscopic repair in the paediatric population. Future research should focus on standardising surgical techniques, rehabilitation protocols, and long-term outcome reporting to strengthen the evidence base. In summary, meniscal repair in young patients represents not only a procedure to restore function but also an investment in lifelong joint health.

## Appendices

PRISMA checklist

**Table 3:** Preferred Reporting Items for Systematic Reviews and Meta-Analyses (PRISMA) 2020 Checklist This checklist documents where each PRISMA 2020 reporting item is addressed in this article. Locations are given by section/subsection headings.

Section/topic	Item #	Checklist item	Location in manuscript
TITLE	1	Identify the report as a systematic review and/or meta-analysis.	Title page; Abstract title
ABSTRACT	2	Provide a structured summary including background, objectives, data sources, eligibility criteria, participants, interventions, study appraisal and synthesis methods, results, limitations, conclusions, and registration.	Structured Abstract
INTRODUCTION	3	Describe the rationale for the review in the context of existing knowledge.	Introduction, paragraphs 1–3
INTRODUCTION	4	Provide an explicit statement of the objectives or questions being addressed.	Introduction, final paragraph
METHODS	5	Indicate if a review protocol exists and where it can be accessed; include registration information.	Methods: Study Design, Registration and Reporting Framework (PROSPERO CRD420251050682)
METHODS	6	Specify all eligibility criteria, including inclusion and exclusion criteria, and rationale.	Methods: Eligibility Criteria
METHODS	7	Describe all information sources (databases, registers, websites, etc.) and dates of last search.	Methods: Search Strategy and Information Sources
METHODS	8	Present full search strategies for at least one database, including any filters and limits used.	Methods: Search Strategy and Information Sources (core terms and limits)
METHODS	9	State the process for selecting studies (screening, eligibility, included in review).	Methods: Study Selection and Data Extraction
METHODS	10	Describe methods of data collection from reports (e.g., piloted forms, independent extraction, duplicate).	Methods: Study Selection and Data Extraction
METHODS	11	List and define all outcomes for which data were sought, including measurement methods.	Methods: Eligibility Criteria; Data Synthesis and Statistical Analysis
METHODS	12	Describe any processes for obtaining or confirming data from study investigators.	Not applicable (all data from published reports)
METHODS	13a	Describe methods used to assess risk of bias of included studies.	Methods: Quality Assessment (MINORS tool)
METHODS	13b	Describe any assessment of risk of bias that may affect cumulative evidence (e.g., publication bias).	Not performed (small number of studies)
METHODS	14	Specify effect measures used for each outcome.	Methods: Data Synthesis and Statistical Analysis
METHODS	15	Describe methods of synthesis including models, measures of heterogeneity, and software used.	Methods: Data Synthesis and Statistical Analysis (random-effects, I²/χ²; single-arm pooling)
METHODS	16	Describe any methods used to explore causes of heterogeneity (subgroup/ sensitivity analyses).	Not performed (limited number of studies)
METHODS	17	Describe any methods used to assess reporting bias due to missing results.	Not performed (insufficient number of studies)
METHODS	18	Describe methods of certainty/quality of evidence assessment.	Methods: Quality Assessment (MINORS)
RESULTS	19	Provide a flow diagram of study selection and numbers screened, assessed, and included.	Results: Study Selection; Figure 1 (PRISMA 2020 Flow Diagram)
RESULTS	20	Present characteristics of included studies.	Results: Study Characteristics and Patient Demographics; Table 1
RESULTS	21	Present risk of bias assessments for each included study.	Results: Quality Assessment Findings; Table 2 (MINORS)
RESULTS	22	For each outcome, present results of individual studies and syntheses, including summary estimates and precision.	Results: Functional Outcomes; Figure 2 (Forest Plots)
RESULTS	23	Present results of investigations of heterogeneity, if performed.	Results: Functional Outcomes (I² and χ² reported)
RESULTS	24	Present results of any assessments of reporting bias.	Not applicable (not performed)
DISCUSSION	25	Provide a general interpretation of the results in the context of other evidence.	Discussion: Main Findings and Clinical Interpretation; Comparison with Previous Literature
DISCUSSION	26	Discuss limitations of the evidence and of the review processes used.	Discussion: Limitations
OTHER	27	Report funding sources and role of funders, and any competing interests.	Declarations: Funding; Conflict of Interest
